# Physico-chemical assessment of torrefied Eurasian pinecones

**DOI:** 10.1186/s13068-020-01840-7

**Published:** 2020-12-07

**Authors:** Alok Dhaundiyal, Divine Atsu, Laszlo Toth

**Affiliations:** 1 Institute of Process Engineering, Szent Istvan University, Godollo, 2100 Hungary; 2 Department of Energy Systems Engineering, Koforidua Technical University, Koforidua, Ghana

**Keywords:** Heat of reaction, Pinecones, Thermal decomposition, Exergy, Torrefaction

## Abstract

**Background:**

Biomass pre-treatment is gaining attention as a standalone process to improve the qualitative aspect of the lignocellulosic material. It has been gaining ground in the power station by replacing the coal with the pre-treated biomass. In this context, this paper enlightens the operating condition of carrying out the torrefaction so that the process can be made relatively more effective. The influence of physico-chemical characteristics on the heat of reaction of pyrolysis reactions, mass loss and temperature regimes are evaluated by thermogravimetry of the pre-treated samples of the pinecone; whereas, the structural transformation in the basic constituents is determined via knowing the fractional change in cellulose, hemicellulose and acid-insoluble lignin contents of the pine cone. The thermogravimetric (TGA) and differential thermal analysis (DTA) were performed to determine the physical as well as the thermal behaviour of the thermally processed biomass. The samples had undergone thermal decomposition at heating rates of 5 °C min^−1^, 10 °C min^−1^ and 15 °C min^−1^. Nitrogen gas was used as a purge gas for the pyrolysis of the pre-treated samples. The volumetric rate of 200 ml min^−1^ was pre-set for the thermal decomposition of the samples at 600 °C; whereas, the selected torrefaction temperature range varied from 210 to 250 °C.

**Results:**

The heat of reaction for the pre-treated samples was found to vary from 1.04 to 1.52 MJ kg^−1^; whereas, it was 0.91–1.54 MJ kg^−1^ for the raw samples. The total annual production cost of processing 3.6 Mg of fuel in a year at a pilot scale was $ 36.72; whereas, the fiscal burden per kilogram of fuel during thermal degradation of the processed fuel was reduced by 0.08–1.5ȼ. The entropy of the system decreased with an increasing ramp rate. The exergetic gain in the system increased by 1–2%. The loss of energy during the energy-intensive processing of the pre-treated fuel was relatively low at a heating rate of 5 °C min^−1^.

**Conclusion:**

By the physico-chemical assessment, it was determined that pinecones required the highest torrefaction temperature and time to provide the upgraded pinecones. It was concluded that the duration of the torrefaction should be at least 15 min for a temperature of 250 °C so that the chemical exergy of the system, energy yield and the energy density of the processed material are qualitatively improved. The volatile and ash contents were noticed to decrease during the torrefaction process. The least fractional change in the volatile content was estimated at 210 °C for a torrefaction time of 15 min; whereas, the ash content was minimum at 210 °C for a torrefaction time of 5 min.

## Background

Energy is essential for society and the development of the industry. However, the ever-increasing demand for the global energy market is mainly met by fossil fuels. Nevertheless, in the coming decades, a paradigm shift in energy policies might be seen due to the increasing demand for energy with the increasing population of the world. An alternative resource should be in the market, which can offset the climate change and can be replenished at a lower cost. It is a premonition of the International Energy Agency that the reliance on oil resources will be at its peak by 2030, and the factors which would influence this prediction are the rate of consumption, exploration of new oil sites, reforms in drilling technology to exploit oil reserves, and ebbs and flows in the oil prices. Thus, the dead reservoir might be revived and will become lucrative in the future.

Moreover, the new policies of the energy market based on fossil fuels are always debriefed by the decision-makers, and the environmentalists as the burning of the conventional fuels magnify the percentage of greenhouse gases in the atmosphere, so there is a need to widen the spectrum of energy sources. According to the economic stance, the imported fossil fuels and their detrimental effect on the ecosystem are in direct conflict with the country’s vested interest. During the oil crisis in the early 1970s, the demand for alternatives to petroleum-based fuels came into the picture. It started the quest of deriving energy through the combustion of plant-based fuels [1]. The popularity of solid biomass for energy generation can only materialise by extending the application of biomass in the energy sector [2]. Currently, the agro-based products, wheat and corn stalk, together with plantation wood; short rotation coppices, willow and poplar, are used as the alternative fuels in the energy sector. In the coming years, the gamut of biomaterials will be widened by bringing the industrial residuals, the mixture of different agriculture and wood waste, and aquatic biomass into the mainstream.

In a similar context, consumption of heterogeneous biomass requires the development of new or improved processing methods so that it can be made compatible with the existing fuel conversion technology. Most of the combustion units require pure biofuel; whereas in some cases, the biomass is co-fired with fossil fuels. Not all the biomasses are necessarily appropriate for all the technologies; therefore, it is crucial to know the class and ranking of the fuel so that the material handling, as well as its transformation into useful energy, can be carried out efficiently. The physical properties of solid biomass vary between fuel type and among fuels of a similar class. Some factors like particle size, ash content, moisture content, and ash melting point, influence the conversion process of the solid biomass. This trade-off between optimal transformation and physical state of biomass can be improved by fuel preparation and upgradation of biomass.

Thermal pre-treatment or Torrefaction is one of such processes which enriches the biomass so that it can compete with different ranks of fuel. The temperature range for the thermal pre-treatment process varies from 200 to 300 °C. It provides the biomass with excellent properties for handling, milling and co-firing with coal in power plants. The market potential for torrefied biomass is considered to be very promising as the final product can be used in power generation units (from power plants to domestic boilers). In Europe’s energy plan (National Renewable Energy Action Plan), emphasis is laid on the reduction of CO_2_ on a massive scale; therefore, it is highly recommended to burn torrefied biomass in power stations in Europe. Torrefaction of feedstock before its usage in a power plant unit reduces the challenges faced during thermo-chemical conversion.

In some experimental studies, it has been found that physico-chemical transformation accompanied by the variation in the fraction of the main components of the biomass happened during the torrefaction process. This process not only expels the moisture content from the biomass but also causes the thermal degradation of cellulose, hemicellulose and lignin in lignocellulosic biomass [3]. It is worth mentioning here that hemicellulose is easily hydrolysed, so it gets affected the most [4]. During the torrefaction of corncobs, it is seen that structural transformations in hemicellulose, cellulose and lignin take place. Xylan has mass loss of 12.2–28.5% during the torrefaction period of 10 min at a temperature range of 220–250 °C; whereas, lignin degradation largely depends on oxygen-containing compounds (phenol, methoxyl, aliphatic alcohol, carbonyl, and ether) and, hence, it decomposes over a wide range of temperature [5].

On the other hand, cellulose is thermally stable at a torrefaction temperature of 220 °C, and the mass loss of 3% takes place during the pre-treatment process [6]. The reason for the high thermal stability at low temperatures is the carbonisation and cross-linking reaction, which makes it mechanically and thermally immune [7]. Due to the variation in the chemical structure of basic polymers of biomass (cellulose, hemicellulose and lignin), the reaction pathways, as well as the thermal decomposition, are different from its parent form [8].

**Fig. 1:**
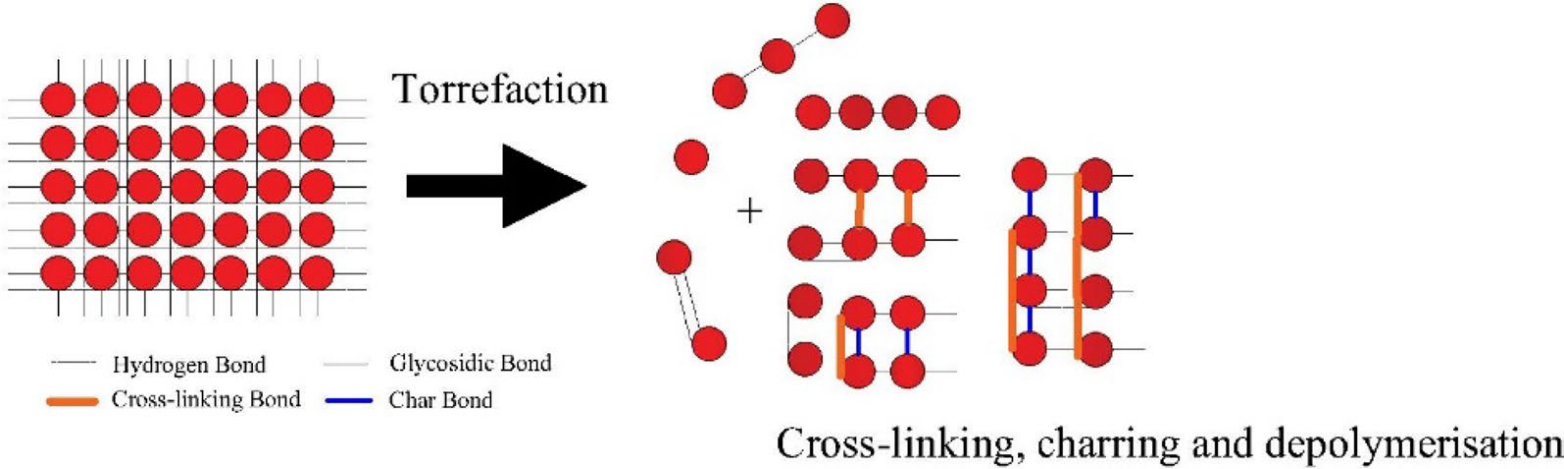
Effect of torrefaction on the lignocellulosic structure

It has been reported that the cleavage of β-*O*-4 linkages liberates the phenolic group from etherified phenolic hydroxyl and consequently, increases the aromaticity of biomass during the torrefaction process [5, 7, 9]. Another study explains that the cleavage of lignin ether bonds and decomposition of carbohydrates promotes the re-condensation reaction to form aromatic C–C and C–H bonds [10–12]. Based on the factual evidence, it is concluded that the thermal treatment leads to depolymerisation of different components. Moreover, the constituent which is highly affected by the heating rate is lignin. The cleavage of the β-*O*-4 bond of lignin begins at 245 °C. Therefore, it becomes necessary not to overlook structural changes during the thermal processing of biomass. Since it changes the reaction pathway of different polymers, the yield of the final products gets influenced by the thermal pre-treatment [13, 14]. Some elementals, as well as physical changes also occur while processing the biomass [3]. It has been found that the produced bio-chars have a lower ratio of H/C and O/C, whereas the grinding ability and hydrophobicity are improved [15, 16]. The emission of CO_2_ and volatile matter during the combustion of the obtained bio-chars is lower than that of the unprocessed biomass [17]. The carbon fraction and the calorific value of torrefied biomass increase at elevated temperature with longer residence time; whereas, it has an opposite effect on the molar ratio of O/H. For better comprehension, a sample of wood generates more solid products than the agricultural residue (bagasse) after the torrefaction process. Moreover, the calorific value and composition of gaseous products (especially CO, CH_4_ and hydrogen carbons) increase with increasing residence time. However, the particle size also influences the heat transfer as well as the residence time. The residence time gets reduced when the rate of heat of transfer to or within the particle is faster than the chemical reaction rate. This implies that solid temperature must be essentially homogenous throughout the reaction; thus, the sole controlling factor would be the intrinsic kinetics only. Therefore, an improvised furnace has been used, and it has been tried to maintain the thermal homogeneity throughout the process. The majority of studies have focussed on microwave-based torrefaction [18, 19], which have a faster rate of heat transfer than the Joule heating system. Moreover, thermal decomposition would be more efficient if the Biot number would be sufficiently much smaller than unity [20]. That makes the torrefaction process rate-limiting and allows a homogenous decomposition throughout the process. However, it is suggested that the nature of biomass also influences the process. It is concluded that the mass yields of hardwood and softwood differ drastically from each other under similar torrefaction conditions [20]. Since xylan is the active component of the hemicellulose of hardwood (80–90%), while it is 15–30% in the case of softwood, therefore, the mass yield is relatively less than that of the softwood. The moisture content of biomass is another important aspect of the mass yield of processed biomass. The samples of corn stover that has different moisture percentage do not have the same dry matter proportion after thermal pre-treatment. It is found that the sample with higher moisture increases the dry matter loss by 10% [21]. There is a high likelihood of correlation between the concentration of water molecules with the rate of hydrolysis during the torrefaction process. It can be concluded from this fact that thermal pre-treatment of biomass is impacted by the varying concentration of oxygen in the biomass [21]. The upgradation of biomass after torrefaction leads to the physico-chemical transformation in the biomass [22]. The transportation and inventory cost of holding biomass waste is also curtailed to some extent [23]. The effect of pre-treatment on the lignocellulose structure is shown in Fig. 1.

The aforementioned factors had encouraged to carry out the upgradation process on those biomasses which are abundant in nature and might be used in a constructive manner rather than creating upheaval in the society [24–27]. The objective of this work is to investigate the physico-chemical changes in the pre-treated pinecones samples. The powder sample of pinecones is examined at different temperature and torrefaction time, and the optimum solution is further analysed by chemical and thermal means. The effect of thermal treatment on the kinetics and heat of reaction are also examined.

## Results and discussion

The analysis is related to the enrichment of forest residues so that it can be introduced into the main energy stream by the bio-industries. Pinecones were thermally processed at different times and temperatures, and it was observed that the torrefaction time of 15 min would be suitable for the thermal pre-treatment. During the physico-chemical testing, it had been seen that the higher temperature profile promoted the mass loss at a uniform rate; whereas, the energy yield, energy density, fixed carbon and chemical exergy increased with increasing temperature and time. Furthermore, the ash content and the volatile matter decreased with increasing temperature. The detailed experimental analysis is divided into two parts: physico-chemical analysis and thermogravimetry of torrefied biomass.

### Physico-chemical analysis

The criteria for selecting the appropriate thermal condition are based on the energy yield, chemical exergy and the energy density of the thermally processed product. However, the decision-making factor might vary with the objective of the bio-industry, so there is no hard and fast rule of selecting a particular condition, but for the sake of the evaluation, higher range of temperature and time was chosen over the lower ones. From a financial point of view of the biomass power plant, it is important to eliminate the fibrous components of a fuel and make it thermally stable. In this way, the energy burden on the ancillary components gets lessened to some extent. From a practical example, a throat-less gasifier causes channelling and bridging for loose biomass; whereas, the same unit is suitable for gasification of dense wood chips. Not only the main unit is affected by the varying physico-chemical characteristics of fuel, but also the functioning of the ancillary components gets hampered. The bearing of the primary blower runs out before its expected lifetime if the tar content drastically increases beyond the permissible limit. Similarly, the energy consumption of the venturi scrubbers and the electrostatic precipitator, and efficiency of the cyclone are severely impacted if the dust content increases with time. Therefore, fuel upgradation is required to overcome the complication that arises during the operation of the power plant.

The basic characteristic of the raw pinecones determined by the physico-chemical analysis shows that ash and volatile contents are proportionally higher than the fixed carbon in pinecones. The energy and bulk densities, as compared to another solid biomass (wood), also are relatively low. The energy consumption during the milling of the unprocessed pinecones is given in Table 1. It is clear from the result that the milling of the crude form requires excessive power for the fuel preparation purposes if the moisture content is very high. The clogged sieve has been shown in Fig. 8e.

**Table 1:** Electrical parameters of 3φ Milling machine

Energy consumption (kW h)	Line voltage, V (V)	Power factor (ɸ)	Milling efficiency (η_g_)	Current I (A)	*Annual expenditure to process pinecones ($)
0.648	387.9145	0.373094	53.34 %	3.041316	36.72

^*^Depends on the electricity rate of the country

The upgrading process was performed at different time and temperature by allowing the milled pinecones to undergo torrefaction in the furnace. The physico-chemical changes along with the power consumption of the furnace at different thermal history are provided in Table 2. It is clear that the physical and chemical characteristics of a material depend on the thermal conditions. However, there is no established relationship between temperature and the physical properties, but there is no doubt that the fuel properties would alter with the torrefaction time and temperature. The magnitude of the chemical exergy, energy density, fixed carbon and ash content increases with the increase in the degree of torrefaction; whereas, the volatile content reduces with it. The optimum values of the energy density and the chemical exergy was obtained at 250 °C for the torrefaction time of 15 min. The lowest value of volatile matter was obtained at 250 °C for the duration of 10 min; whereas, the ash content was found to be 0.47% when the processed pinecones was thermally pre-treated at 210 °C for a period of 5 min. Similar behaviour has been observed while processing the rick husk and Licorice residue at 210–240 °C [28, 29]. This happened since hemicellulose plays a crucial role in the rearrangement of hydrogen and oxygen molecules in the main structure of the biomass [30]. The P-value of the experimental results is provided in Table 3. It is clear from the statistical analysis that the duration of the torrefaction has a significant effect on the ash content, energy density and mass yield of the processed pinecones; whereas, the torrefaction temperature has a higher impact on the fixed carbon and volatile matter of the processed pinecones. Alternatively, the power is assumed to be invariant throughout the process.

**Table 2:** Physico-chemical characteristic and the annual processing cost of pinecones

Torrefaction time (min.)	T (°C)	F.C (%)	Ash content (%)	V.M (%)	Energy density (GJ m^−3^)	Chemical exergy *ε* (MJ kg^−1^)	Mass input (g)	Mass output (g)	M.Y%	Average energy consumption of the improvised pilot unit (kW-h)
5	210	92.46	0.47	3.86	5.62	20.91	2.96	2.92	95.61%	0.16
	220	94.00	0.63	2.20	6.04	21.28	5.06	4.80		
	230	79.12	0.64	17.17	6.13	21.43	6.49	6.18		
	240	84.94	0.78	10.30	6.12	20.95	6.76	6.54		
	250	82.16	0.94	13.83	6.09	22.09	9.16	8.47		
10	210	84.15	1.94	10.83	6	21.78	7.42	6.73	88%	0.066
	220	94.33	0.89	1.076	6.25	22.58	6.35	6.05		
	230	86.79	1.15	9.06	6.16	22.37	4.59	3.75		
	240	87.76	1.58	6.68	6.35	22.93	5.77	5.13		
	250	94.31	1.36	1.25	6.60	23.26	5.44	4.70		
15	210	94.15	0.96	1.36	5.98	21.58	6.88	6.3	87%	0.18
	220	92.34	1.52	2.96	6.20	22.02	5.75	4.9		
	230	84.46	1.23	11.32	6.72	24.16	4.81	4.11		
	240	85.07	2.50	8.45	7.22	24.46	5.55	4.98		
	250	91.33	1.31	4.04	8.44	28.37	5.41	4.6		
Total							82.99	75.56	91%	0.40
Raw material	–	10.27	1.41	80.35	5.95	20.40	155	82.99	53.54%	0.648

**Table 3:** Statistical analysis of the obtained parameters (significance level = 0.10)

Parameter	Factor	*P*-value
F.C	Duration of torrefaction	0.6
	Torrefaction temperature	0.09
V.M	Duration of Torrefaction	0.4
	Torrefaction temperature	0.1
Ash Content	Duration of torrefaction	0.02
	Torrefaction temperature	0.6
Energy density	Duration of torrefaction	0.08
	Torrefaction temperature	0.2
Mass yield	Duration of torrefaction	0.008
	Torrefaction temperature	0.50
Power consumption	Duration of torrefaction	0.50
	Torrefaction temperature	0.68

**Fig. 2:**
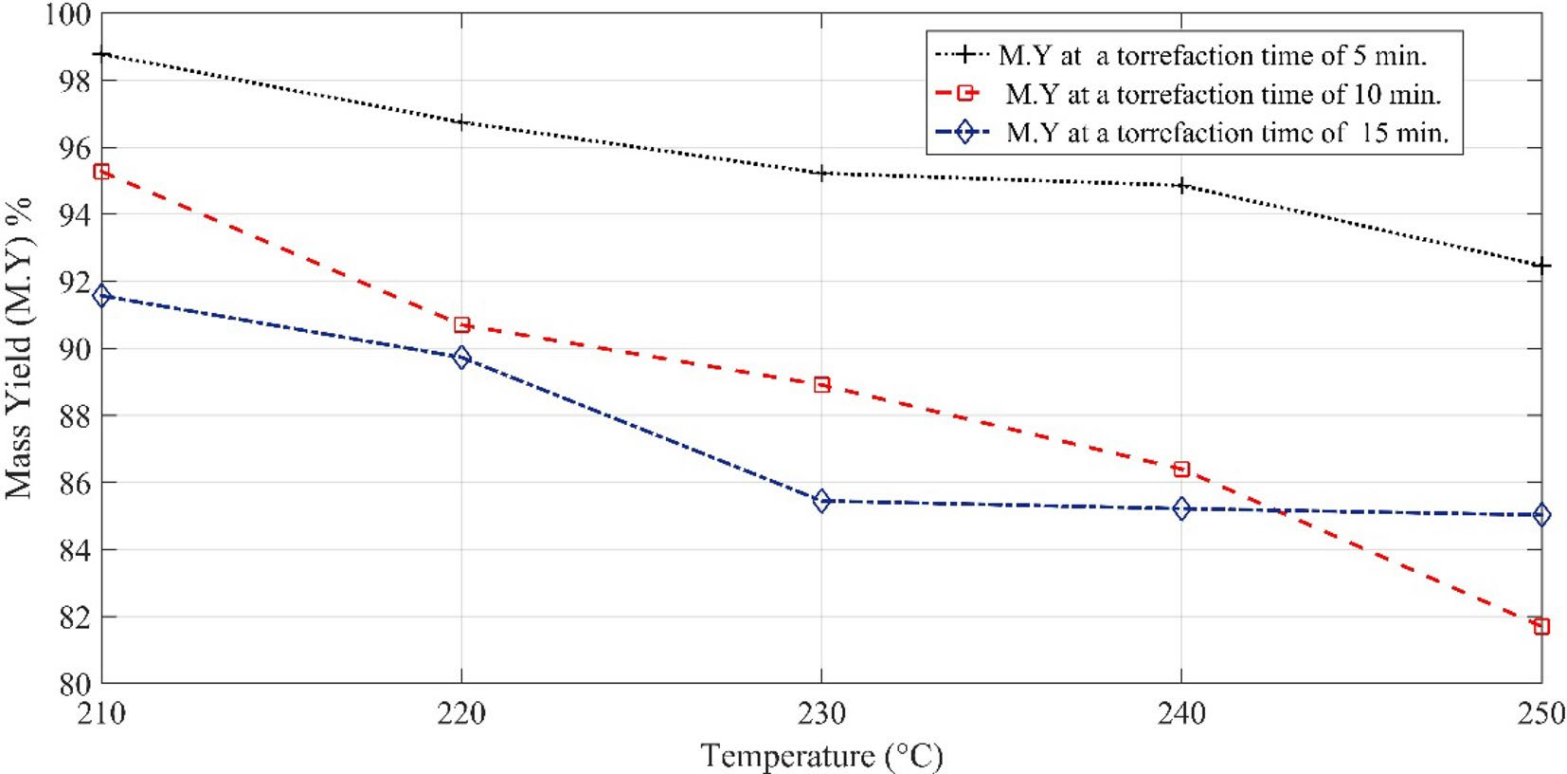
Mass yield of the pinecones at different torrefaction time

**Fig. 3:**
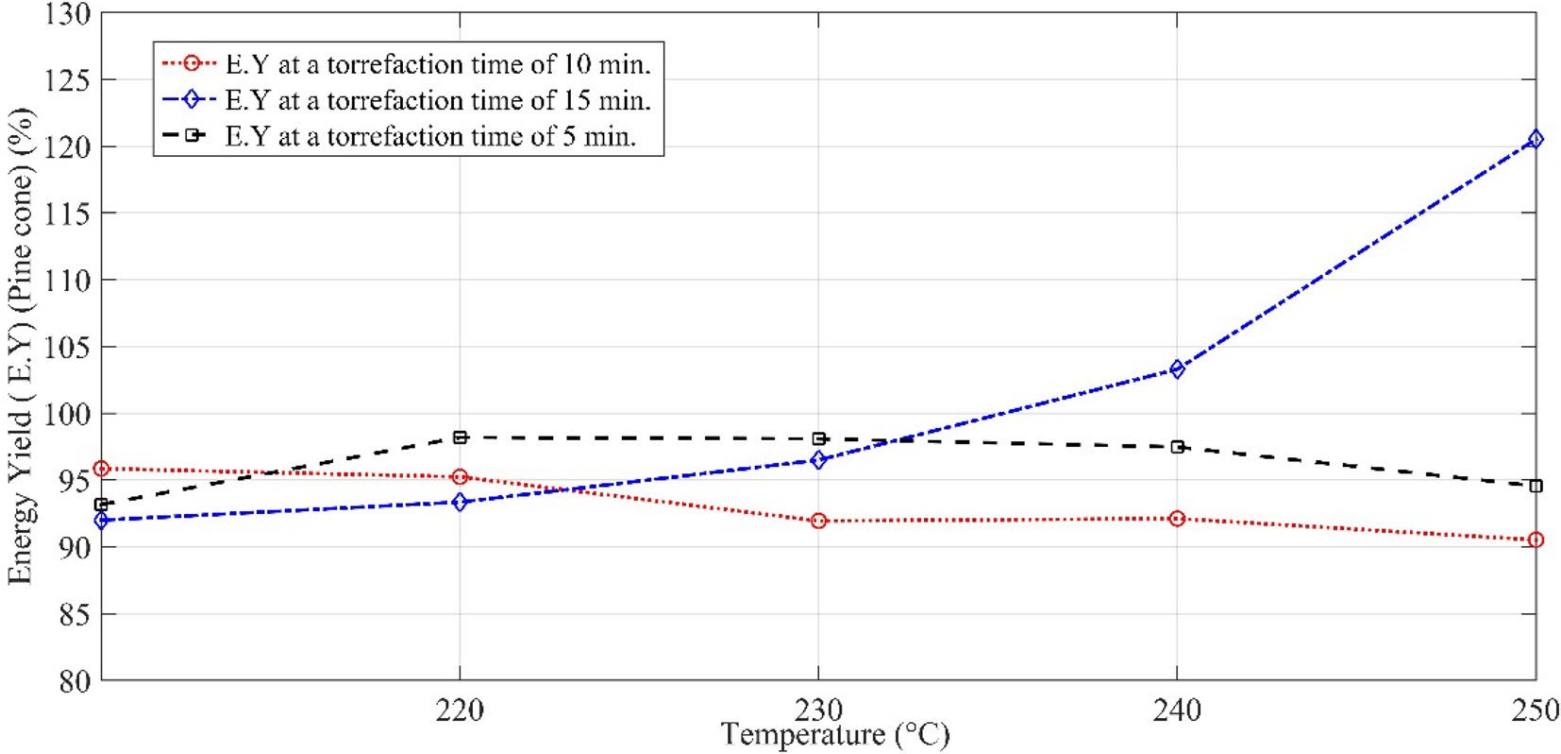
Energy yield of the pinecones at the varying processing time

Furthermore, hemicellulose has ample carboxyl groups, and the decarboxylation reaction triggers as the temperature of the furnace increases. With the decarboxylation of hemicellulose, the oxygen-containing compounds such as water, CO_2_, CO and oxygenated organics get liberated, and thus, the molar ratio of the oxygen and carbon (O/C) decreases. Consequently, the removal of water and oxygen increases the heating value of biomass, and in this way, a physical rearrangement of molecules is ascribed to the qualitative improvement of the biomass. Moreover, the inability of the OH group to make hydrogen bond imparts hydrophobicity to the thermally pre-treated biomass. The variation of mass yield with temperature is illustrated in Fig. 2. It is clear from Fig. 2 that the mass loss has a linear relationship with the change in the temperature of the system. At a constant temperature of 210 °C, the mass yield of pinecones for pre-treatment duration of 15 min processing is 6.5% lower than the mass yield of pinecones processed for 5 min; whereas, it is 3.7% for the torrefaction period of 10 min. This mass loss gap increases as the torrefaction temperature increase with time. On the other hand, the energy yield, which is shown in Fig. 3, drastically increases with increasing temperature and time. The lower temperature range for a longer duration of torrefaction does not bring out any appreciable change in the energy yield, even though it reduces the energy yield by 3.15%.

**Fig. 4:**
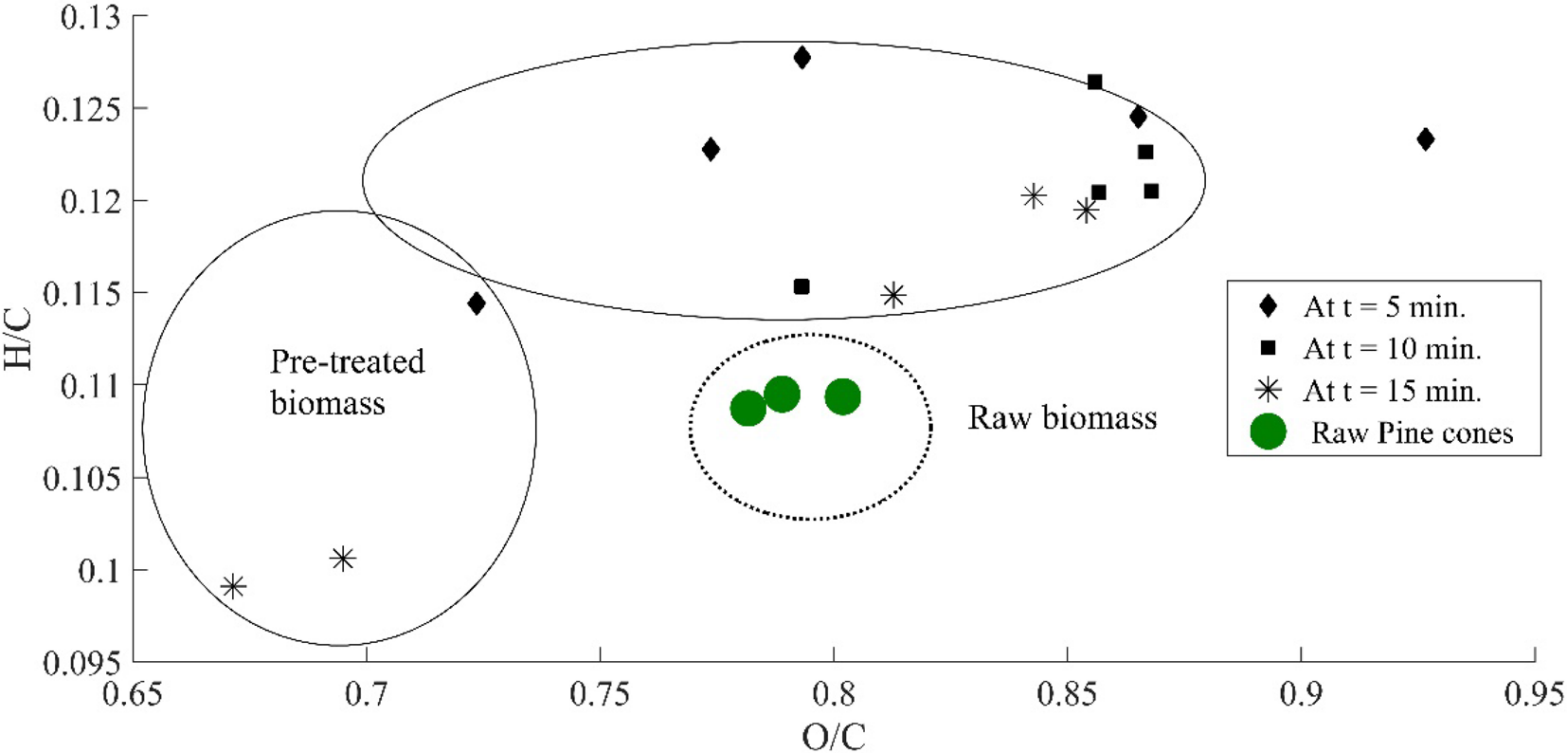
Van Krevelen diagram of the raw and pre-treated pinecones

**Fig. 5:**
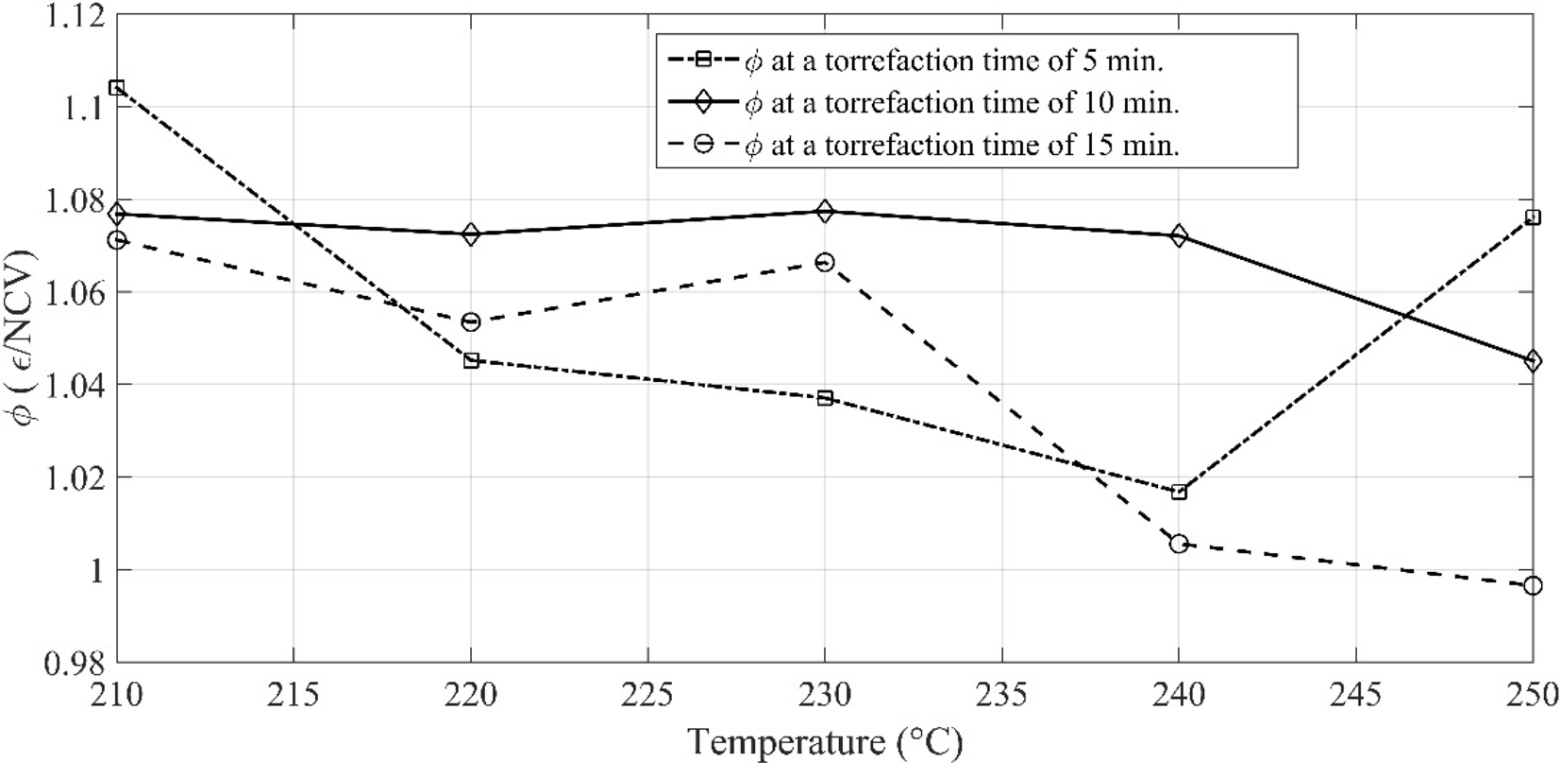
The ratio of *φ* (exergy-NCV) at different pre-treatment time

At a constant temperature of 250 °C, the energy yield for the torrefaction time of 15 min is 26% higher than that of the torrefaction time of 5 min; whereas, it is − 5.2% for a processing time of 10 min. So, it is not necessary that the torrefaction for a prolonged time might provide an optimistic energy yield. Therefore, it is essential to find the optimum solution for a particular fuel. It also infers that the gain in energy yield depends on the initial state of the pinecones. In other words, high moisture content of biomass increases the dry matter loss. It shows the concentration of water molecules is highly correlated to desorption during the torrefaction process [21]. It is also possible that the optimum solution for pinecones shall not provide the same results for another woody biomass. The plot of the molar ratios is shown in Fig. 4. The molar ratios of H/C and O/C decrease with increasing degree of torrefaction. However, the molar ratio of H/C is relatively high as compared to O/C. The reason is the deprivation of O_2_ in the flame front causing the formation of carbon monoxide; whereas, the remaining fraction of the carbon dioxide reacts with carbon to reduce into carbon monoxide. The overall heat of reaction is endothermic; therefore, the mass loss increases with temperature and time. This reaction is also called the Boudouard reaction. During the torrefaction process, some of the carbon gets drained away with the volatile gas in the form of soot particles. Another aspect of torrefaction is described by the chemical exergy of the pre-treated feedstock, which is depicted by Fig. 5. The ratio of the chemical exergy to net calorific value (NCV) (*φ*) is also seen as a linear function of temperature. It is seen that the chemical exergy of the system decreases as the torrefaction temperature, and the time frame increase. It defines whether the exergy of the reactive system in relation to the NCV is increasing or decreasing. The *φ* for the pre-treated samples of pinecones varies from 0.99 to 1.07, whereas it is 1.023 for the unprocessed pinecones. The value of φ for coal and wood lies in the domain of 1.06–1.10 and 1.15–1.30, respectively [31]. It is clear from this fact that some of the pre-treated samples are having similar traits as the different ranks of coal exhibit.

The percentage fraction of cellulose, hemicellulose, and lignin in a pre-treated and raw material is tabulated in Table 4. There is an 8.8% decrease in the mass fraction of cellulose during torrefaction of pinecones at 250 °C for 15 min, whereas it is 43.63% for hemicellulose. On the other hand, the acid-insoluble lignin fraction is increased by 88.8%, with an increase in torrefaction time and temperature. A similar trend is seen while thermally treating lignocellulosic components. Moreover, the aromaticity of lignin is also increased during the thermal processing [5–7, 10]. The reason is that the cleavage of β-*O*-4 linkages initiate when the thermal processing is done above 245 °C [13]. The sudden increase in aromaticity of the pre-treated pinecones shows the decomposition of the lignin ether bonds, which re-condense to form 15 aromatic C–C and C–H bonds [10, 12]. It can be concluded that thermal treatment leads to the depolymerisation of different components and affects the yield of the end products.

**Table 4:** Composition of the main constituents of pinecones at 250 °C for the processing period of 15 min

Substrate	Cellulose %	Hemicellulose %	Lignin (AIL) %
Raw feedstock	32.91	22.92	30
Pre-treated	30	12.92	56.65

### Thermogravimetry of pre-treated pinecones

Thermal degradation of processed and the raw pinecones is performed at the heating rate of 5 °C min^−1^, 10 °C min^−1^, and 15 °C min^−1^ by the thermogravimetric analyzer. The temperature range for the pyrolysis of the materials is kept between 32 and 600 °C. The thermal profile is selected to be linear with time. In addition, the heat of reaction during the pyrolysis process is computed by differential thermal analysis of both the materials. The reference material Alumina powder having a heat of fusion of 1.092 kJ g^−1^ is chosen for the measurement purposes. The decomposition process is carried out under a nitrogen environment. The sign notations ‘−’ and ‘+’ denote decrease and increase in the magnitude of that physical parameter, respectively.

**Fig. 6:**
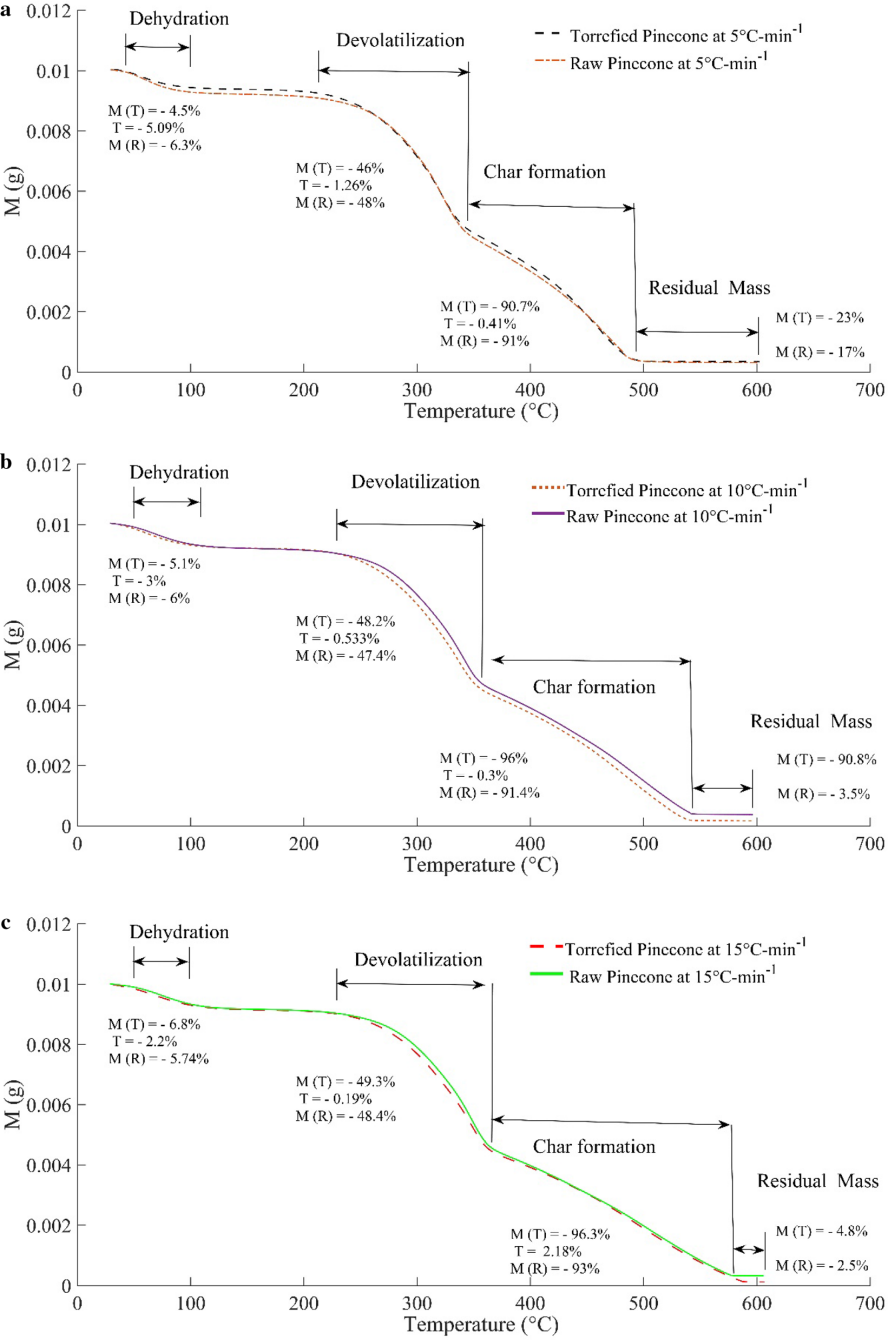
Thermogravimetric analysis (TGA) of the raw and processed pinecones at different heating rates (**a** TGA at 5 °C-min^−1^, **b** TGA at 10 °C min^−1^, **c** TGA at 15 °C min^−1^)

The illustration of the mass loss during pyrolysis of the pre-treated and raw pinecones at different heating rates is shown in Fig. 6. Degradation of mass is demarcated by the three different stages: dehydration, devolatilisation and char formation. The boundaries of these regimes related to the raw material are marked up by the lines and the shift in the temperature and mass conversion is examined while investigating thermogravimetric behaviour of the raw material. At the onset of the pyrolysis process, the fractional change of mass of a thermally pre-treated sample during the dehydration stage is lower than that of the raw material by 28%. Moreover, the shift in the temperature scale has got to be seen during the dehydration phase. The temperature range over which evaporation of the moisture takes place is found to be reduced by 5.09%. On the other hand, the domain of devolatilisation for the torrefied pinecones is shrunken by 0.19 at the heating rate of 5 °C min^−1^ (Fig. 6a). In a similar context, the char formation reaction is relatively discouraged, and the scale of the temperature is shrunken by 0.41%. The major proportion of the mass loss in the processed pinecones is encountered during the char formation (90.7%) and devolatilisation (46%); however, thermal immunity has been seen in relation to the raw material.

The residual mass loss variation, in the context of raw pinecones, is also increased by 35% during the thermal decomposition of the torrefied pinecones. A similar trend is to be seen at higher heating profiles (Fig. 6b). At a thermal heating rate of 10 °C min^−1^, the mass loss gap between the raw and the processed pinecones is decreased by 15%. Unlike the shrinking domain of the devolatilisation at a lower heating rate, the domain of the devolatilisation is relatively expanded by a margin of 58%. Consequently, the range of temperature required during the char formation is also relatively increased by 27% with an increase in the heating rate. As the mass loss of the pre-treated pinecones is not very predominant at a lower heating rate, but with the change in thermal profile, the mass reduction rate of the pre-treated pinecones supersedes the mass decomposition rate of the raw pinecones. The acceleration in the mass loss during the devolatilisation phase of the pre-treated pinecones is 5% higher than that of the raw cones. Similarly, the char formation at higher heating rates is also increased by 6.6% as compared to the char formation at 5 °C min^−1^. However, with the increasing heating rate, the mass loss during ash formation is also increased with a huge margin of 90%, which is merely 23% at a heating rate of 5 °C  min^−1^. To ascertain this fact, the heating rate is increased by 50% (Fig. 6c) and it is found that the temperature gap between the pre-treated pine cones and the raw cones is curtailed and even in some stages, it exceeded the temperature range of the raw material. The mass loss variation during the dehydration stage is 13.3% when the heating rate is increased from 5 °C min^−1^ to 10 °C min^−1^; whereas, the mass loss gap during the dehydration process is increased to 33.33% as the heating rate is increased by the next 5 °C min^−1^. However, this gap is reduced to 2.2%, which is nearly 5% at a heating rate of 10 °C min^−1^ during devolatilisation of the pre-treated sample at 15 °C min^−1^. The mass variation between the raw pinecones and the pre-treated pinecones is 20% higher than the mass variation at 10 °C min^−1^. A similar trend in the increase of the mass variation with the heating rate is seen during the devolatilisation stage. But the relative char production is essentially the same at a heating rate of 10 °C min^−1^ and 15 °C min^−1^. Moreover, the char production of raw pinecones is relatively increased at higher heating rates. The range of temperature during the char production is appreciably higher than that of the domain of char at 10 °C min^−1^. The variation in the mass loss of the pre-treated pinecones during the formation of the residual mass is drastically decreased by 95%. It can be concluded that the char formation during the thermal decomposition of the pre-treated pinecones gets saturated with the heating rate; whereas, the residual mass has a varying nature which is difficult to predict since it depends on the extent of the char formation. If the thermal properties of the pinecones at higher temperature regimes keep on changing with the heating rate, the residual mass will be affected. Another reason is that the residence time of volatile in the solid matrix affects the autocatalytic reactions, which in turn influence the char and gas yields.

The domain of devolatilisation that changes with the temperature promotes the mass loss at a higher heating rate. However, the mass loss of the raw material during devolatilisation has a marginal variation of 1.25–2.1%. On the other hand, the mass decomposition at the beginning of the thermal degradation is relatively increasing in the case of the pre-treated pinecones, whereas the effect of the heating profile on the raw material depicts the opposite pattern. The reason for deviation in the thermal properties of the pre-treated pinecones is the physical variation in the structure of the biomass. The direction of flow of the heat related to the grain orientation affects the heat transfer across the specimen. Due to the internal failure of the material while undergoing the thermal treatment, the local porosity and the permeability influence the flow of the fluid across the material, which results in the altered heating characteristic of the material. An experiment has been conducted on the wooden dowel, and it has been observed that the drastic fluctuation of the pressure at the centre of the dowel leads to severe structural failures, such as longitude channelling and surface cracking. It has been suggested that these surface cracks influence the heat transportation across the material [29]. Despite having a constant rate of heat transfer, these cracks promote the heat to flow more quickly to the interior and hence, affect the fluid flow inside. Another reason is the anisotropy of the material due to the grain orientation. The permeability for flow of volatiles along the grain is 10000 times more than that across the grain. In a similar manner, thermal resistance offered along the grain is one-half of the thermal resistance across the grain [30]. It has been reported that the fluid flow across the grains ascribes the secondary pyrolysis reactions which depend on the resistance time of volatile inside the cellulose matrix.

**Fig. 7:**
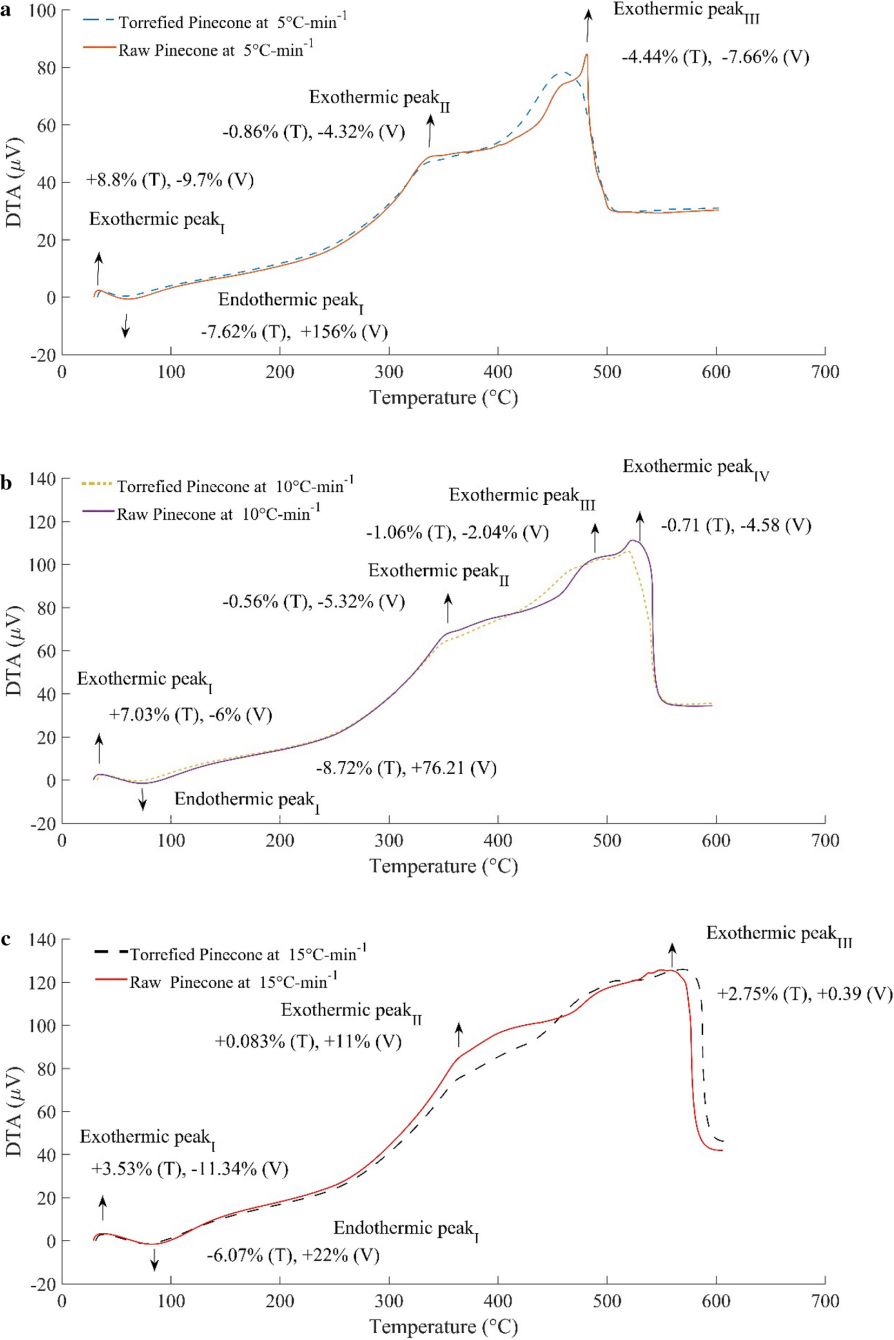
Differential thermal analysis (DTA) of torrefied and the raw pinecones at a varying thermal history (**a** DTA at 5 °C min^−1^, **b** DTA at 10 °C-min^−1^, **c** DTA at 15 °C min^−1^)

The impact of these physical parameters can be seen in the distribution of the heat of reaction among the different stages of pyrolysis. However, the kinetics of pyrolysis also plays a crucial role in determining the end product yield. For large size particles, the temperature gradient across is considerably high, which in turn promotes the reaction to be driven via a convection model rather than conduction alone. Since the value of the Biot number is greater than unity, the heat transfer rate is dominated by the rate of reaction. The heat of reaction is determined with the help of Eq. 5. Differential thermal analysis (DTA) of the pre-treated pinecones and the raw material is shown in Fig. 7. The upward direction or voltage gain denotes the exothermic reactions; whereas, the voltage drop or the downward trend implies the endothermic nature of the reactions. Similar to TGA, the relative change in the positions of local maxima or minima of the pre-treated pinecones with respect to the raw pinecones is demarcated in a DTA plot.

The calibration factor and the Monte Carlo technique [32] are used to determine the area under the peaks. It is clear from Fig. 7a that the first exothermic peak (I) region for the torrefied pinecones is shifted right; whereas, the temperature for the exothermic region (I) is 8.8% higher than that of the exothermic region (I) of the raw pinecones. The voltage drop corresponding to the power of the system, of 9.7%, is seen at the beginning of pyrolysis. The endothermic region (I) of the DTA curve for the pre-treated biomass has seen a reduction in the temperature range during dehydration of the pre-treated pinecones. It implies that the heat of reaction (Endothermic) during the dehydration is reduced by a margin of 7.62%; whereas, the energy absorbed by the system is reduced by 99.14%. Also, the heat of reaction during devolatilisation (Exothermic peak II) is relatively reduced by 2.55%. On the other hand, there is a small variation (around 0.15%) in the heat of reaction between the raw and the pre-treated pinecones as they undergo the char formation at a heating rate of 5 °C min^−1^.

Similarly, the change in the positions of the local maxima and minima over a given range of temperatures has been observed at higher heating rates. Except for the change in the position of peaks, the distribution of the heat of reaction among the different stages of pyrolysis is rearranged by the formation of the intermediate products, which results in an additional number of peaks during the process. This phenomenon is observed at the heating rates of 10 °C min^−1^ and 15 °C min^−1^, where the overall exothermic heat of reaction is distributed among the different peaks. The additional peak shares 12% of the total heat of reaction while processing the raw pinecones at heating a rate of 10 °C min^−1^; whereas, it increases in case of the decomposition of the pre-treated pinecones at a heating rate of 15 °C min^−1^ to 19.2%. The relative variation of the temperature range during the dehydration phase (Endothermic peak I) is reduced by 30% as the heating rate increases from 5 °C min^−1^ to 15 °C min^−1^. The range of temperature over which the heat of devolatilisation varies is increased by 34.88–110% when the heating rate increases from 5 °C min^−1^ to 15 °C min^−1^; whereas, this range is further increased from 84 to 162% during the char formation at the higher heating rates. With the increasing heating rates, the energy released during the charring process to the ambience is 50–52% lesser than the released energy at 5 °C min^−1^.

Similarly, the reduction in energy of the system during devolatilisation at the higher heating rates varies from 8 to 23%. The overall heat of reaction at the higher ramping rates decreases by 0.6–41% for the raw pinecones, and 0.8–32% for the pre-treated pinecones. It can be concluded that the variation of the reaction heat in the thermally processed material is relatively low as compared to the raw form of it. The energy burden on the system increases at higher thermal profiles. In the context of the involvement of energy-intensive processes during the thermal decomposition and the overall heat of reaction, the lower heating rates for pyrolysis of the pre-treated pinecones is preferable to the higher heating rates. The full detail of the heat of reaction along with energy reduction is summarised in Table 5. The entropy of the system decreases with the increasing temperature and heating rates. The net exergy input, as well as the exergetic efficiency at a lower heating rate, are found to be higher than that of higher heating rate (Table 6). The fiscal burden (Table 6) during the pyrolysis is also seen to be reduced while pyrolyzing the pre-treated pinecones at a lower heating rate. The pre-treatment process reduces the indirect expenses required to tackle the energy-intensive process. The total saving per kg of fuel varies from 0.08ȼto 1.5ȼ. The process is found to be very effective for the large capacity biomass plants, where the consumption varies from 1 to 2 Mg.

**Table 5:** Heat of reaction (Δ*H*) during thermal decomposition of the pre-treated pinecone and the raw pinecone

Reaction regime	Δ*H* (kJ kg^−1^) Unprocessed pinecones			Δ*H* (kJ kg^−1^) Torrified pinecones		
	5 °C min^−1^	10 °C min^−1^	15 °C min^−1^	5 °C min^−1^	10 °C min^−1^	15 °C min^−1^
Exothermic (I), kJ kg^−1^	− 3.83	− 4.52	− 6.74	–	− 4.88	− 5.59
Temperature range (I) °C	29–50	28–56	28–64	–	32–60	31–62
Endothermic(I), kJ kg^−1^	1.88	3.08	2.36	0.016	0.66	1.77
Temperature range (I) °C	50–72	56–90.72	65–98.35	32–32.4	60–76.14	62–93
Exothermic (II), kJ kg^−1^	− 697.85	− 468.35	− 455	− 680	− 520	− 426
Temperature range (II) °C	72–335	90.78–351	98–364	32.5–333	36–352.1	93–361.36
Exothermic (III), kJ kg^−1^	–	− 182.42	–	–	–	− 200
Temperature range (II) °C	–	350–483	–	–	–	361.4–508
Exothermic (IV), kJ kg^−1^	− 839.26	− 877	− 450	− 838	− 882	− 412.35
Temperature range (IV) °C	335–602	351–596	363–606	334–603	352.2–596	508–607
Net heat of reactions (MJ/kg)	− 1.54	− 1.53	− 0.91	− 1.52	− 1.40	− 1.04
Energy saving (%)	0	0	0	99.14%	78.57%	25%

**Table 6:** Thermodynamic and the cost parameters related to the pyrolysis process

Reaction regime	ΔG (kJ kg^−1^) Unprocessed pinecones			ΔG (kJ kg^−1^) Torrified pinecones		
	5 °C min^−1^	10 °C min^−1^	15 °C min^−1^	5 °C min^−1^	10 °C min^−1^	15 °C min^−1^
Exerogonic (I)	− 3.82	− 4.50	− 6.72	–	− 4.86	− 5.57
Endergonic(II)	1.87	3.06	2.35	0.02	0.66	1.76
Exerogonic (II)	− 695.42	− 466.71	− 453.41	− 677.62	− 518.18	− 424.51
Exerogonic (III)	–	− 181.78	–	–	–	− 199.30
Exerogonic (IV)	− 836.32	− 873.93	− 448.42	− 835.067	− 878.91	− 410.91
Entropy of a system (kJ kg^−1^ K^−1^)	0.066	0.067	0.036	− 0.0098	− 0.0094	− 0.0069
Exergetic gain	0	0	0	2.06%	1.02%	1.6%
Capital saving per kg of fuel*	0	0	0	1.5ȼ	0.24ȼ	0.08ȼ

* It depends on the energy policy of a country

The peculiar behaviour of the thermally processed biomass is due to the extent of the secondary pyrolysis reactions, which are highly influenced by the relative direction of heating with respect to the grain orientation. It has been experimentally found that the overall heat of reaction at higher heat fluxes is exothermic in nature, which is more significant when heating is perpendicular to the grain orientation than when it is parallel [33]. In another study, it has been reported that the exothermic nature of the reaction is strongly influenced by the lignin content of biomass [30]. The reason behind this behaviour is the thermal conductivity of biomass which changes during the thermal pre-treatment process. It has been observed that the thermal conductivity of the biomass along its grain orientation is twice that across it. It is to be noted that the role of the secondary pyrolysis is necessary to take into consideration when the thermal conductivity across the grain direction is considered in the pyrolysis modelling [30]. The moisture content of biomass must also be considered as one of the reasons for the variation in the heat of reaction since the solid internal temperature history of the biomass and the intra-particle energy balance are influenced by the evolution of water (endothermic). According to the multi-step kinetic scheme, the char catalysed decomposition reactions account for the dependency of the exothermic pyrolysis reaction and the secondary char reactions on the apparent residence time of volatile [30]. The increase in intra-particle residence time of volatile at lower heating rate encourages the char formation reactions, which are also highly exothermic in nature and account for large char yield. The residence time dependency on the heating rates is one of the most common assertions to define the extent of the secondary reaction and the shift in the reaction pathways [34].

In a nutshell, it can be concluded that the overall improvement of the torrefaction process relies on temperature and the ramping rate. The torrefaction has been conducted at the isothermal condition, and the obtained results comply with the results obtained in literature [35–37]. During torrefaction of wood, pine, birch and bagasse, it has been found that the calorific value of the biomass increases with both temperature and time, while the yield of biomass decreases with time [35]. However, it cannot be the case if the system is being operated at the quasi-static operating range. Though the calorific value increases with temperature, it has been found that there is no direct relationship between time and temperature. It depends more on the moisture content of the raw feedstock than temperature–time relation [21]. Similar results have been found when the wheat straw has been pre-treated, and it has been noticed that the calorific value increased by 20% with 5% reduction in oxygen. But with an increase in the calorific value, the mass loss also increases; therefore, there must be a critical operation temperature/time when the results get optimum in terms of energy and mass.

## Conclusion

The study pivots around the methodology adopted to carry out the torrefaction to optimise the energy content of biomass and reduce the ash content at a minimal mass loss. The increase in ash content and the presence of inorganic catalyst in the ash go hand in hand with the torrefaction temperature; therefore, such analysis becomes more indispensable to have an in-depth detailing of the process. The fast torrefaction during the microwave heating increases the thermal lag across the specimen; therefore, the rate of heating must be either slow or constant during the thermal pre-treatment.

The pre-treatment process of pinecones has been performed in a modified furnace at the quasi-static condition. The processed pinecones have been investigated chemically, physically, thermally and on the economic basis as well. Thermal analysis is carried out to ascertain whether the thermal behaviour of the biomass after the pre-treatment process shows a deviation in the reaction of heat, mass loss or not. The total energy consumption cost required to process 5 kg pinecones for a year is found to be $ 36.72.

By the physico-chemical assessment, it has been determined that pinecones required the highest torrefaction temperature and time to provide the upgraded pinecones. It has been concluded that the duration of the torrefaction should be, at least, 15 min for a temperature of 250 °C, so that the chemical exergy of the system, energy yield and the energy density of the processed material are qualitatively improved. Though, the degree of torrefaction is subjected to the physical attributes of the biomass being used in the furnace. The volatile and ash contents are found to decrease during the torrefaction process. The least fractional change in the volatile content is estimated at 210 °C for torrefaction time of 15 min; whereas, the ash content is minimum at 210 °C for torrefaction time of 5 min. On the other hand, the fixed carbon, the energy density and the chemical exergy of the pre-treated pinecones increase with increasing torrefaction temperature and duration of the process.

The molar ratio of O/C is minimum at a torrefaction temperature of 250 °C for 15 min. Both the O/C and H/C are observed to be decreased with the increasing temperature of the furnace. The average energy consumption of the modified unit for 15 min is 0.18 kW h. The fraction of cellulose in the processed pinecones was found to be decreased by 8.8%; whereas, the fraction of hemicellulose reduced to 12.92% during the torrefaction process. The percentage fraction of acid-insoluble lignin in the torrefied sample is found to be increased by 88.8%. The ash content at the selected torrefaction condition decreased by 7.1%, which is a new finding of this analysis since in the majority of torrefaction, it has been seen that the ash content increases with increasing torrefaction temperature. On the other hand, the energy density is increased by 46% and the chemical exergy, by 39%. Similarly, a drastic change in the fixed carbon fraction is observed.

According to the thermogravimetric analysis, the processed pinecones are found to be thermally more stable than the raw pinecones. The domain of char formation increases at the elevated heating profile; whereas, the domain of devolatilisation reduces with an increasing heating rate. The mass loss during the devolatilisation stage increased with increasing heating rates for the pre-treated pinecones. A similar trend had been noticed during charring of the pre-treated pinecones. The exergetic gain in the system increased by 1–2%. The loss of energy during the energy-intensive processing of the pre-treated fuel is relatively low at a heating rate of 5 °C min^−1^. The heat of reaction for the pre-treated samples varies from 1.04 to 1.52 MJ  kg^−1^; whereas, it is 0.91–1.54 MJ kg^−1^ for the raw samples. The energy-intensive process is least at 5 °C min^−1^; whereas, it relatively increased with the heating rate. Thermal decomposition of the samples is accompanied by enthalpy change of the system (number of peak areas change with time and temperatures). The total annual production cost of processing 3.6 Mg of fuel in a year at a pilot scale is $ 36.72; whereas, the fiscal burden per kilogram of fuel during thermal degradation of the processed fuel is reduced by 0.08–1.5ȼ. The entropy of the system decreased with an increasing ramp rate.

## Materials and methods

### Mechanical/thermal processing of pinecones

**Fig.8:**
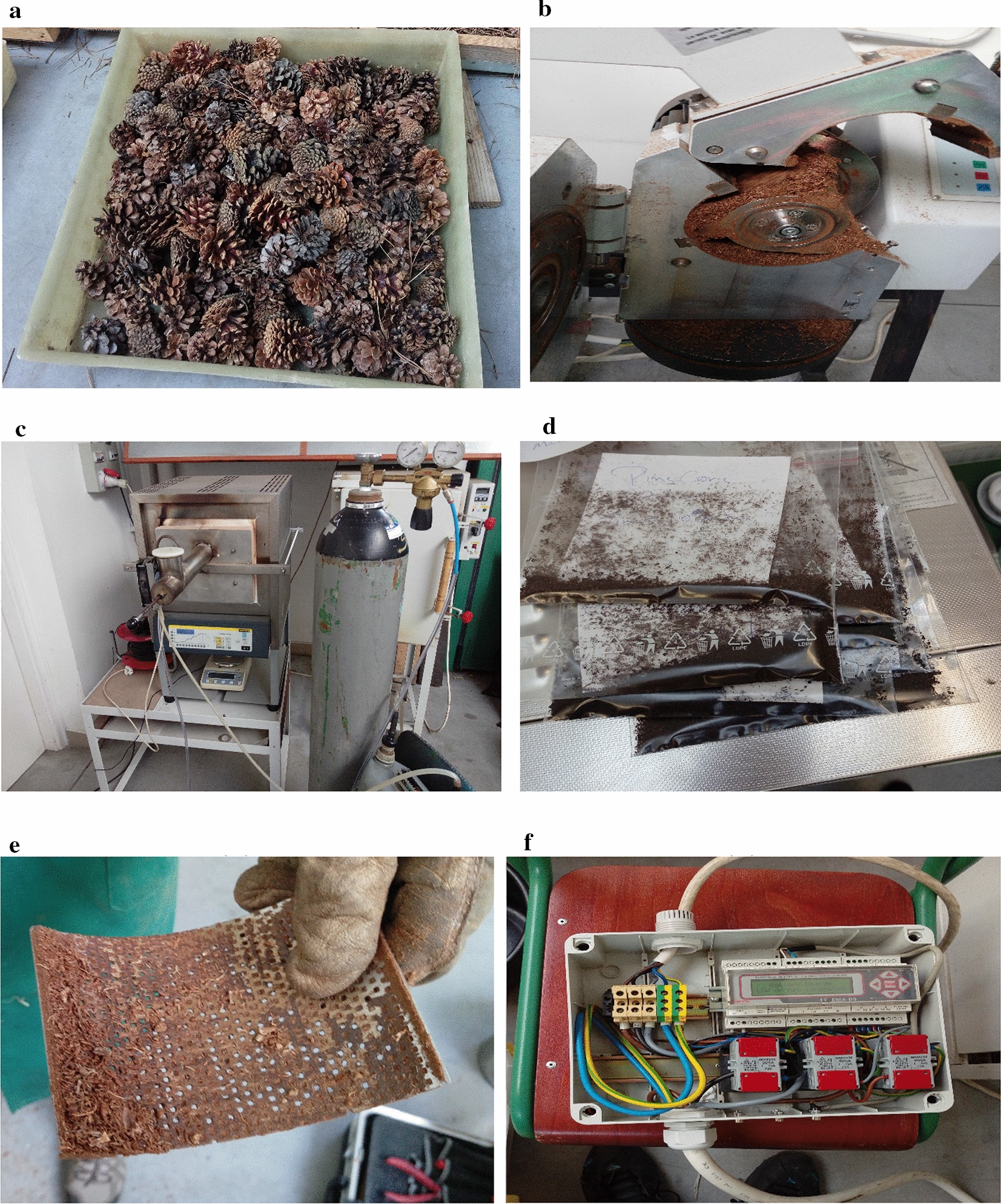
Milling and torrefaction of pinecone (**a** pinecone storage, **b** milling process, **c** torrefaction in the improvised furnace, **d** the clogged sieve of diameter 2 mm, **e** multi-function analyser)

The feedstock was collected from Pest county of Hungary. Thermal processing and material preparation were performed at the National Agriculture Research and Innovation Centre, Hungary. For fuel preparation, pinecones (Fig. 8a) were allowed to pass through a sieve of 1 mm (Fig. 8e) in the milling machine. The thermal treatment process (Torrefaction) was carried out on the basis of temperature and time. The selected domain of torrefaction temperature was 210–250 °C; whereas, the torrefaction period chosen for the given material was varied from 5 to 15 min. The mechanically processed pinecones had undergone torrefaction in the modified furnace. The power consumption of the milling machine was measured with the help of the modified version of the multi-function analyser (Fig. 8f); whereas, the single-phase energy logger was used to measure the energy consumption of the furnace. The digitally programmed furnace (Fig. 8c) retrofitted with a modified lid was provided with two ports for the flow of an inert gas (Nitrogen is used in this case), and a cylinder with a plunger for injecting the feedstock into the receptacle. Before bringing the furnace into operation, it was programmed for a particular thermal profile. The cylindrical section jutted out of the lid was used to hold the samples. Once the desired temperature was attained by the furnace, the feedstock was fed to a vessel placed inside the furnace by the plunger. A fan retrofitted to the jutted part of the lid was employed so that feedstock might not be affected by the elevating temperature of the furnace.

The vessel was mechanically connected to the weighing machine by a link so that the mass variation of feedstock could be monitored. The pressure of nitrogen gas used for experimental was 1.5 bar. The purge rate of 700 mL min^−1^ was pre-set to scavenge air from the chamber as well as the cylinder. Consequently, the ingression of oxygen is deterred to prevent the surface oxidation of feedstock. The surface oxidation accelerates the internal heat and mass transfer when the temperature and superficial velocity are increased [38]; thus, an appreciable drop in the solid and the energy yield is noticed, which is not favourable for the current study [38, 39]. It is expected that the decomposition reaction is controlled by heat and mass transfer rather than the rate of reaction. However, the quenching effect and the energy consumption of the furnace increases drastically due to the effects of the surplus flow of the inert gas; therefore, the flow of nitrogen must be regulated as per the degree of torrefaction required for a particular biomass.

**Fig.9:**
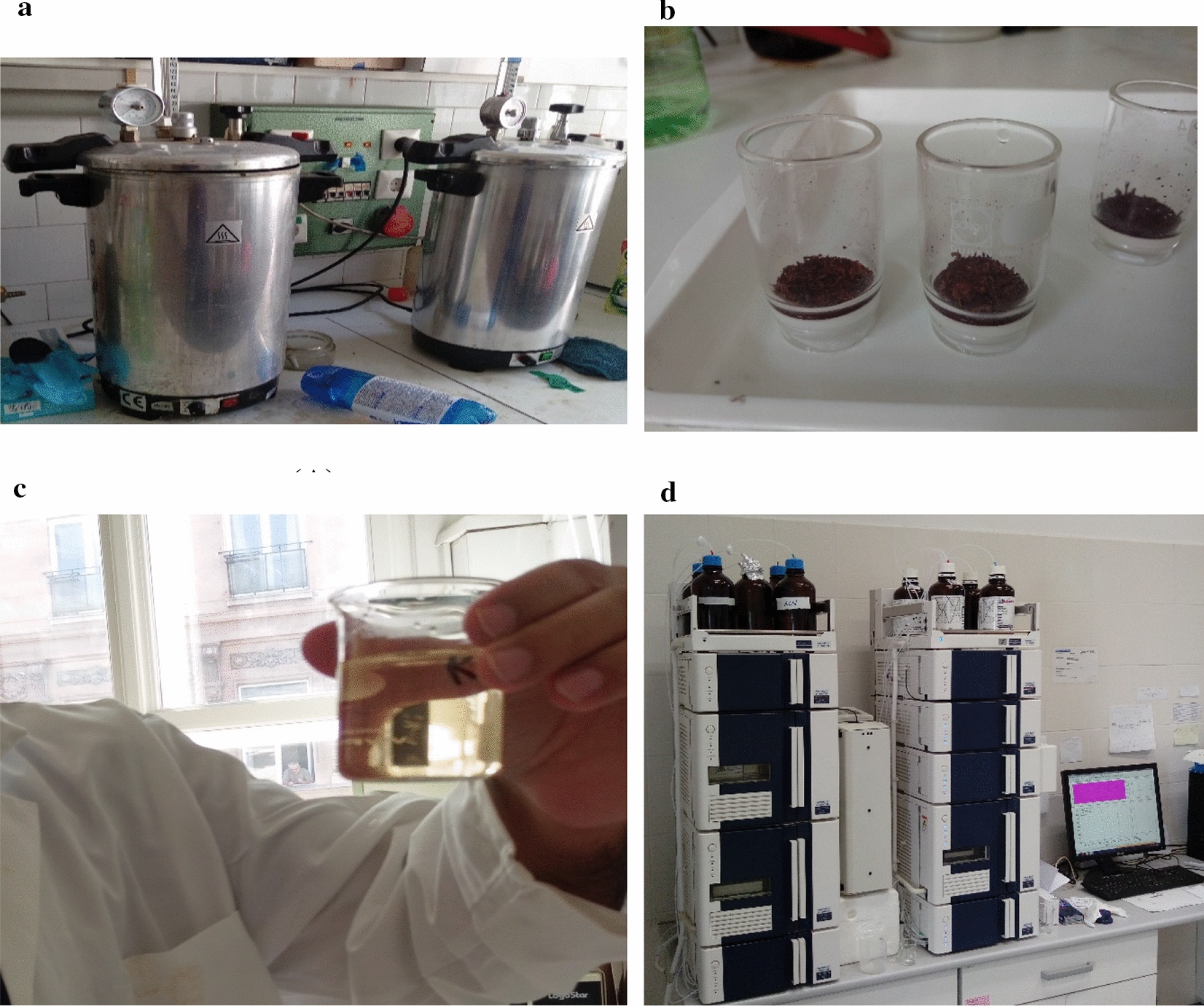
The chemical treatment of the pinecones (**a** autoclave, **b** acid-insoluble lignin, **c** liquid fraction for HPLC, **d** high performance liquid chromatography)

Once the furnace reaches the desired temperature, the sample is introduced in the chamber so that the effect of the heating rate does not influence the physical characteristics of the feedstock in an unexpected way. Since the physical and chemical properties of biomass (fixed carbon, volatile matter and calorific value) vary linearly with temperature [40] and the mass diffusivity also increases with the increasing ramping rate of the temperature which results in the formation of the reactive gases at the interface of the solid and the inert gas, so it is necessary to maintain the mass variation during the formation of the boundary layer. However, the diffusion of gases is relatively faster, and it influences the reaction rate of the pyrolysis. Moreover, the vapour pressure of oxygen is low, and the ingress of it triggers the surface oxidation reaction. Therefore, the thermal parameters of the furnace must not exceed the operational condition. Figure 9 illustrates the equipment and the raw material used for the experiment.

After the completion of the torrefaction process, the processed samples were further investigated in an oxygen bomb calorimeter. Before initiating the measurement of calorific value, the calorimeter was calibrated using the benzoic tablets. The samples were tested in a stainless-steel vessel. The permissible operating pressure of the calorimeter was 23 MPa, and the oxygen pressure should not exceed 4 MPa in the vessel. The water inside the cell was allowed to heat at 25–30 °C. The permissible limit of energy provided to the decomposition vessel should not be more than 40 kJ. The IKA WERKE C2000 interface software was used to evaluate the heating value of the processed pine needles. The elemental composition of the pre-treated samples was calculated by the Vario MACRO Elementar. In the beginning, the analyser was heated up to 1200 °C for 30 min. Before measuring the elemental composition, the analyser was calibrated by the standard birch leaf so that it can be examined whether the unit is functioning properly or not. Once the procedure was finished, the torrefied samples were encapsulated in a tin foil with tungsten (VI) oxide (WO_3_), which acts as a reagent in the chemical reaction and facilitates the oxidation of the samples. Once reaching the pre-combustion temperature, the samples were rejected through the rotating disk mounted on the top of the analyser. The volumetric rate of oxygen was maintained throughout the process to assist the catalytic combustion; whereas, helium gas was used as a carrier gas. The role of the carrier gas (He) is to ensure that the products of combustion are properly carried away to different reduction columns. The reduction columns are in-between the combustion chamber and the signal-processing unit. The elements of the gas are separated by purge or trap chromatography. As the components of the gas get separated, each of them is individually detected by a thermal conductivity detector (TCD). The products of combustion are absorbed in a sequence. Though, nitrogen does not pass through the reduction columns. TCD creates the electric pulse that is proportional to the concentration of the elementary components of the biomass. In addition, the post-combustion in the CHN(S) analyser assists in the complete oxidation of the biomass samples. The material used for the reduction purpose was tungsten. The elemental composition of the pinecones is given in Table 7. It is to be noted that the European Standard EN 15104:2011 (“Solid biofuels—Determination of total content of carbon, hydrogen and nitrogen—Instrumental methods”) is used for the computational purpose. Oxygen content in the samples was measured using the Eq. (1). The chemical composition of the pinecones and the physical parameters are provided in Tables 7 and 8.

1 $${\text{O}}\left( \% \right) = 100 - \left( {{\text{C}}\left( \% \right) + {\text{H}}\left( \% \right) + {\text{N}}\left( \% \right) + {\text{S}}\left( \% \right) + {\text{Ash}}\left( \% \right)} \right)\left( {{\text{dry basis}}} \right).$$

**Table 7:** Ultimate analysis of Pinecone (wet basis)

C%	H%	N%	S%	O%	HHV^*^ (MJ kg^−1^)	Ash %
48.62	5.310	0.943	0.103	38.45	20.14	1.41

**Table 8:** Proximate analysis of the pinecone with some physical parameters

F.C %	V.C%	M.C%	Bulk density (kg m^−3^)	Energy density (GJ m^−3^)
10.27	80.35	7.96	295.6	5.95

The mass yield (M.Y) of the pre-treated sample is evaluated by [41, 42]

2 $${\text{M.Y}} \left( \% \right) = \left( {\frac{{M_{t} }}{{M_{b} }}} \right) \times 100.$$

The energy yield (E.Y) of the processed sample of the pine needles is measured by the following expressions [41, 42]:

3 $${\text{E.Y}} \left( \% \right) = {\text{M.Y}} \times \left( {\frac{{{\text{HHV}}_{t} }}{{{\text{HHV}}_{b} }}} \right) \times 100.$$

${\text{HHV}}_{t}$ and ${\text{HV}}_{b}$ represent higher heating value of the pre-treated (torrefied) and the raw samples, respectively.

The ratio $\varphi =\left(\frac{\varepsilon }{\text{NCV}}\right)$ of the chemical exergy ($\varepsilon )$ to the net calorific value (NCV) is computed by the Eq. 4 [31].

4 $$\varphi = \frac{{[1.0438 + 0.1882\left( \frac{H}{C} \right) - 0.2509 \left( { 1 + 0.7256\left( \frac{H}{c} \right) + 0.0383 \left( \frac{N}{C} \right)} \right]}}{{1 - 0.3035\left( \frac{O}{C} \right)}}\left( {{\text{Valid for}}0.667 < \frac{O}{C} < 2.67} \right).$$

The thermal analysis was performed at the Indian Instrumentation Centre, Indian Institute of Technology, Roorkee, UK, India. To distinguish the torrefied samples from the raw feedstock, the pre-treated samples of the pine needles were analysed at different heating rates. The volumetric flow rate of the nitrogen used for the thermogravimetric purpose was 200 mL min^−1^. The reference material used for the differential thermal analysis was Alumina powder. The horizontal thermo-balance was used for the analysis purpose. The objective of the horizontal thermo-balance is to avert the likelihood of the buoyant effect by the nitrogen gas. The comprehensive details on the balance beam are provided in the literature [43]. The heating rates of 5 °C min^−1^, 10 °C min^−1^ and 15 °C min^−1^ were used for the thermal decomposition. The temperature range of the heating chamber was allowed to vary from 35 to 600 °C. The duration of thermal analysis was 60 min, and the weight of the reference material used for differential thermal analysis (DTA) was 10 mg. The thermocouple type ‘R’ was considered to measure the temperature of the furnace. The heat of reaction was estimated with the help of the differential thermal analysis (DTA) [43]. Equation (5) was used to compute the heat of reaction; whereas, the calibration factor was evaluated by the peak area of the reference material.

The calibration factors for the thermally pre-treated pinecones sample at the heating rate of 5 °C min^−1^, 10 °C min^−1^ and 15 °C min^−1^ are 0.1427 mW μV^−1^, 0.1547 mW μV^−1^ and 0.1628 mW μV^−1^, respectively.

5 $$\Delta H = \pm K. {\text{peak area of DTA}}.$$

#### Chemical treatment of pinecones

The fractional change in the main constituents of the pinecones during the torrefaction process was computed with the help of the National Renewable Energy Laboratory (NREL) method to determine structural carbohydrates and lignin in the biomass [7]. The dry matter was calculated before chemically treating the samples with 72% concentrated H_2_SO_4_. The minimum sample mass used for measurement was 0.5 g. Thereafter, the measured sample was treated with 2.5 mL of sulphuric acid in a 100 mL bottle for two hours. Once the process was completed, 75 mL of distilled water was added in the same graduated bottle. Thereafter, it was kept inside the vertical autoclave for the next one more hour (Fig. 9a). The autoclave had been filled with distilled water up to the level of the base plate. The pressure valve was left open until the temperature of the autoclave reaches 60 °C. It is to be noted that the water level should not be high, nor low, else the safety might be jeopardised, and the temperature and the pressure in the autoclave must not exceed 121 °C and 1 bar, respectively.

The height and the diameter of the pressure chamber are 350 mm and 260 mm, respectively, whereas the volumetric capacity of the vessel is 18 L that can withstand the maximum pressure of 2.4 bar. Meanwhile, the dried sintered glass filter from air heating dryer (105 °C) was placed inside the desiccator so that the ingression of moisture might not affect the measurement process. Once the autoclaving process was finished, the bottle was carefully removed from the chamber. Before the filtration process, the weight of the empty filter was measured. A vacuum flask was used to filter the autoclaved solution with the help of an aspirator, washer and the sintered glass filter. Upon finishing the filtration process, the non-filtered part gets settled in the sintered glass (Fig. 9(B)). The same sintered glass filter was left for overnight drying in the same air heating dryer. On the other hand, the percolated solution (Fig. 9c) was separated through the side arm of the vacuum flask for the HPLC testing. The acid-insoluble lignin can be measured from the following equations.

After it gets dried, the acid-insoluble part (Eqs. 6 and 7) is measured as follows:

6 $${\text{ODW}}_{s} = \frac{{\left( {M_{a} \times \%\, {\text{total solid}}} \right)}}{100}.$$

ODW_s_ is the oven dry weight, *M*_*a*_ is the mass of a dried sample

$$\% {\text{AIR}} = \frac{{\left( {M_{{{\text{fa}}}} - M_{f} } \right)}}{{{\text{ODW}}_{s} }} \times 100,$$

where AIR is the acid-insoluble residue, *M*_fa_ is the total mass of filter and the acid-insoluble residue, *M*_f_ is the mass of filter

7 $${\text{AIL}} \% = \left( {{\text{AIR}} - {\text{Ash}} \left( {{\text{dry basis}}} \right)} \right) \times 100,$$

where AIL is the acid-insoluble lignin.

The HPLC includes the column oven, autosampler and pump as a unit; however, the function of each sub-unit is different. The measured solution was injected into the mobile solvent by a hypodermic syringe. The UV–VIS detector was employed to detect and determine the relative fraction of cellulose, hemicellulose and lignin in the sample. As a light source, a Xenon flash lamp was used to create the collimated light, which gets diffracted from the mobile phase and the change in the wavelength is detected by a detector diode. The wavelength range of Hitachi Chromaster 5430 varies from 190–900 nm. The HPLC grade water filtered with a 0.2 μm membrane filter was chosen for the mobile phase. The purge rate of the solvent was pre-set to 0.6 mL min^−1^. Before mixing the solvent with the sample, the solvents were allowed to be degassed so that it might not cause complexity during the elution process. The solvents get mixed at the solvent mixing valve before passing through a guard column, where the impurities get eliminated so that the C18 column (Nucleosil NH_2_, 250 × 4.6 mm, USA) might not be clogged. The guard column chosen for the experimental varies in length from 20 to 100 mm and it has a diameter of 4.6 mm. The equipment used during the chemical treatment is illustrated in Fig. 9. The list of ancillaries, along with manufacturer details, is provided in Table 9.

**Table 9:** List of ancillaries, manufacturers and the consumable materials

Ancillaries	Manufacturer
Energy Logger 4000	Voltcraft, Hirschau
Electrical Multifunction Analyser EMA-D9	EMA
Furnace	Nabertherm GmbH
Heavy duty cutting Mill SM2000	Retsch GmbH
Weighing machine	Mettler Toledo
Thermogravimetry, SII 6300 EXSTAR	Seiko Instruments Inc
C2000 Oxygen Bomb Calorimeter	IKA-WERKE, Staufen,
Elementar (CHNS)	Vario MACRO cube
Vertical Autoclave, Certoclav Classic	Certoclav Steriliser Gmbh,
HPLC Chromaster 5430	Hitachi
Tungsten (VI) Oxide, WO_3_	Americanelements
Birch leaf Standard (136621)	Elementalmicroanalysis
C18 columns	Nucleosil

## Data Availability

All data generated or analysed during this study are included in this article.
